# Improved Models of Human Endometrial Organoids Based on Hydrogels from Decellularized Endometrium

**DOI:** 10.3390/jpm11060504

**Published:** 2021-06-03

**Authors:** Emilio Francés-Herrero, Elena Juárez-Barber, Hannes Campo, Sara López-Martínez, Lucía de Miguel-Gómez, Amparo Faus, Antonio Pellicer, Hortensia Ferrero, Irene Cervelló

**Affiliations:** 1 Department of Pediatrics, Obstetrics and Gynecology, Faculty of Medicine and Odontology, Universitat de València, 46010 Valencia, Spain; emilio.frances@ivirma.com (E.F.-H.); lucia.demiguel@ivirma.com (L.d.M.-G.); 2 Fundación Instituto Valenciano de Infertilidad (FIVI), Instituto de Investigación Sanitaria La Fe, 46026 Valencia, Spain; elena.juarez@ivirma.com (E.J.-B.); hannes.campo@ivirma.com (H.C.); sara.lopezma@ivirma.com (S.L.-M.); amparo.faus@ivirma.com (A.F.); hortensia.ferrero@ivirma.com (H.F.); 3 Department of Obstetrics and Gynecology, Feinberg School of Medicine, Northwestern University, Chicago, IL 60611, USA; 4 IVIRMA, 00197 Rome, Italy; apellicer@ivirma.com

**Keywords:** endometrium, organoids, decellularization, ECM hydrogel, proliferation

## Abstract

Organoids are three-dimensional (3D) multicellular tissue models that mimic their corresponding in vivo tissue. Successful efforts have derived organoids from primary tissues such as intestine, liver, and pancreas. For human uterine endometrium, the recent generation of 3D structures from primary endometrial cells is inspiring new studies of this important tissue using precise preclinical models. To improve on these 3D models, we decellularized pig endometrium containing tissue-specific extracellular matrix and generated a hydrogel (EndoECM). Next, we derived three lines of human endometrial organoids and cultured them in optimal and suboptimal culture expansion media with or without EndoECM (0.01 mg/mL) as a soluble additive. We characterized the resultant organoids to verify their epithelial origin, long-term chromosomal stability, and stemness properties. Lastly, we determined their proliferation potential under different culture conditions using proliferation rates and immunohistochemical methods. Our results demonstrate the importance of a bioactive environment for the maintenance and proliferation of human endometrial organoids.

## 1. Introduction

The endometrium is the innermost layer of the uterus and is highly regenerative, playing a key role in supporting embryo implantation and pregnancy [1]. Disorders affecting the endometrium typically result in infertility [2,3,4]. Such disorders include endometriosis, which involves the growth of endometrial tissue outside the uterine cavity [5]; adenomyosis, the presence of endometrial glands and stroma within the myometrium [6]; Asherman’s syndrome, the presence of intrauterine and/or intracervical adhesions [7]; and endometrial atrophy, characterized by a nonproliferating endometrium [8]. These conditions are the most common infertility-causing endometrial pathologies, but their etiology is not fully understood, which hinders the development of reliable and optimized treatments. Well-established models of endometrial conditions are needed because, while animal models are often used to simulate many types of gynecological pathologies [9], translation to humans is challenging due to anatomic and physiological differences among species. Two-dimensional (2D) culture of human endometrial cells (i.e., established lines or derived from primary cultures) does not model many important aspects of the native endometrial microenvironment, such as secretions, autocrine/paracrine interactions, and three-dimensional (3D) conformation. In vitro 2D culture of primary human cells has other disadvantages, including difficulty maintaining long-term cultures and loss of cellular phenotype [10]. Consequently, 3D culture systems are valuable emerging alternatives in the study of healthy and pathological tissues and organs.

Organoids are now popular models for both the normal biological function of tissues and disease states. Organoids are self-forming 3D multicellular in vitro tissue platforms that mimic the organ from which they are derived [11]. Three-dimensional constructs have been generated from different organs, such as the intestine [12], kidney [13], and endometrium. In 2017, two groups described the generation of genetically stable and ovarian-hormone-responsive endometrial organoids from glandular epithelium [14,15]. One group later reported the derivation of organoids from patients with different endometrial pathologies (endometriosis and endometrial cancer) [16].

Despite the advantages of organoids over traditional culture methodologies, they are unsuitable for studying the female reproductive tract because they lack stromal cells and therefore do not provide the reciprocal interaction with epithelial cells that contributes to proliferation, differentiation, and decidualization [17]. Indeed, organoids do not reproduce key features, molecular pathways, and cellular functions needed to adequately model the native uterine microenvironment [18]. The inability to model in vivo tissue characteristics such as signaling between cells and their extracellular matrix (ECM) also prevents organoids from reliably recapitulating in vivo tissue diversity [19]. Further, endometrial organoid development requires the use of commercially available extracellular matrices as scaffolds, such as Matrigel [20]. The tumor origin of this matrix, as well as its inability to reproduce the original conditions of the tissue, underscores the need to search for new materials that reliably reproduce in vivo tissue conditions.

Bioengineering methods used in modeling mammalian tissues include decellularizing tissues and organs to generate high-quality ECM scaffolds that support in vitro cell culture [21,22,23,24] and contain structural proteins, growth factors, and other signaling molecules involved in cellular processes such as homeostasis and tissue repair [25]. Concerning the gynecological field, different forms of decellularized ECM are used to enhance uterine repair [26,27] or optimize in vitro embryo culture [28,29], supporting their use in reproductive medicine research. Recently, our group developed a hypoimmunogenic endometrial ECM hydrogel supporting in vitro culture of human endometrial cells and improving the proliferation rate of endometrial stem cells compared with standard matrices such as collagen and Matrigel [30].

Given the need for physiologically relevant experimental models in reproductive medicine, we sought to evaluate ECM hydrogels derived from decellularized endometrium in preparing and improving current human endometrial organoid culture systems. Our starting hypothesis was that supplementing organoid culture medium with solubilized endometrial ECM (EndoECM) hydrogels that mimic the in vivo microenvironment could have a beneficial effect on organoid development, allowing epithelial cells to bind and interact with soluble ECM tissue specific molecules. After generating and exposing human endometrial organoids to tissue-specific ECM hydrogels, we found that they recapitulated the in vivo phenotype of endometrial glands, maintained their chromosomal stability, and had increased proliferation rates. They also expressed typical markers of endometrial epithelial progenitor cells. Our findings support that mimicking the native microenvironment creates a favorable system for endometrial organoids.

## 2. Materials and Methods

### 2.1. Study Design

**Figure 1:**
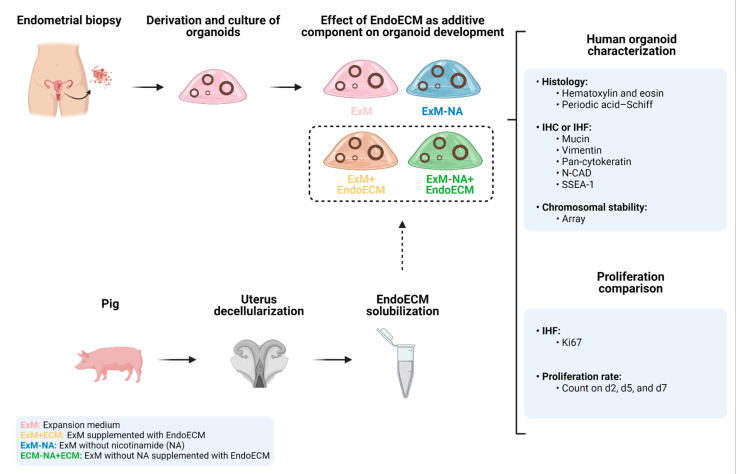
Experimental design. Human endometrial organoids were cultured under four different experimental conditions (ExM, ExM+EndoECM, ExM-NA, ExM-NA+EndoECM) and evaluated microscopically, histologically, immunohistochemically, and for chromosomal stability. Abbreviations: EndoECM, endometrial extracellular matrix; IHC: immunohistochemistry; IHF: immunohistofluorescence; N-CAD: N-cadherin; SSEA-1: stage-specific embryonic antigen-1; ExM: expansion medium; NA; nicotinamide. Created with BioRender.com.

Three human endometrial biopsies were obtained from oocyte donors. The glandular fraction was used to establish organoid lines under standard culture conditions (Figure 1). After four passages (p), organoids were exposed for 7 days (p5) to four different culture conditions: standard (expansion medium; ExM), suboptimal (without the key component nicotinamide; ExM-NA), and EndoECM-hydrogel-supplemented (ExM+EndoECM; ExM-NA+EndoECM) media. Organoids were cultured into Matrigel and EndoECM was used as an additive component in the culture medium. Glandular epithelial marker expression (pan-cytokeratin), absence of stromal components (vimentin), presence of mucopolysaccharide secretions (MUC-1 and periodic acid–Schiff (PAS) staining), stemness characteristics (SSEA-1 and N-cadherin), and cell proliferation rate (Ki67) were histologically and immunohistochemically assessed on day 7. Proliferation rates on days 2, 5, and 7 were compared between the different conditions by counting approaches. Additionally, long-term chromosomal stability of early (p3) and late (p8) passage organoids exposed to ExM was evaluated using a genomic hybridization array. The chromosomal stability of p5 organoids exposed to ExM+EndoECM medium was also analyzed.

### 2.2. Organoid Development from Endometrial Biopsies

This study was approved by the Institutional Review Board of IVI Foundation (1706-FIVI-053-IC, Valencia, Spain). Endometrial biopsies (*n* = 3) were obtained from hormonally stimulated healthy oocyte donors on the day of the oocyte retrieval of controlled ovarian stimulation (COS) cycles undergoing laparoscopy at IVI Valencia. All women were aged 18–33 years, with a body mass index between 20 and 25 kg/m^2^, regular menstrual cycles (21–35 days), and proliferative endometrium. Biopsies were processed at the IVI Foundation as previously described [14]. Tissue was washed in DMEM/F-12 without phenol red (Gibco^TM^, Invitrogen, Paisley, UK, 11039021) and fragmented into 0.5–1 mm^3^ pieces using scalpels. Fragments were enzymatically digested in 15 mL RPMI 1640 medium (Gibco^TM^, Invitrogen, Paisley, UK, 21875034) with 50 U/mL Dispase II (Sigma-Aldrich, St. Louis, MO, USA, D4693) into 10 mM NaAc–5 mM CaAc solution, 4 mg/mL collagenase V (Sigma-Aldrich, St. Louis, MO, USA, C9263), and 10% newborn calf serum (Biowest, Nuaillé, France, S0750) with gentle stirring for 20 min at 37 °C. Digestion was stopped by adding 30 mL RPMI 1640 medium, and the container was swirled. After 2 min, supernatant was passed through 100 μm cell sieves (Corning^TM^, Tewksbury, MA, USA, 431752) three times and washed with RPMI 1640 medium. Sieves were inverted and backwashed with 12 mL advanced DMEM/F12 (Gibco^TM^, Invitrogen, Paisley, UK, 12634010) medium to retrieve retained glandular elements. After centrifugation (500 g for 5 min), the pellet was resuspended in 15% advanced DMEM/F12 and 85% ice-cold Matrigel (Corning™, Bedford, MA, USA, 354234). An amount of 20 μL drops were plated into 48-well plates (Corning^TM^, Kennebunk, ME, USA, 3548), allowed to set at 37 °C, and cultured in organoid expansion medium (ExM). ExM contains advanced DMEM/F12; N2 supplement 1X (Gibco^TM^, Life Technologies, Grand Island, NY, USA, 17502048); B27 supplement minus vitamin A 1X (Gibco^TM^, Life Technologies, Grand Island, NY, USA, 12587010); Primocin, 100 μg/mL (InvivoGen, Toulouse, France, ant-pm-1); N-acetyl L-cysteine, 1.25 mM (Sigma-Aldrich, St. Louis, MO, USA, A9165); L-glutamine, 2 mM (Sigma-Aldrich, St. Louis, MO, USA, G7513); recombinant human EGF, 50 ng/mL (PeproTech, Cranbury, NJ, USA, AF-100-15); recombinant human Noggin, 100 ng/mL (PeproTech, Cranbury, NJ, USA, 120-10c); recombinant human R-Spondin-1, 500 ng/mL (PeproTech, Cranbury, NJ, USA, 120-38); recombinant human FGF-10, 100 ng/mL (PeproTech, Cranbury, NJ, USA, 100-26); recombinant human HGF, 50 ng/mL (PeproTech, Cranbury, NJ, USA, 100-39); ALK-4, -5, -7 inhibitors; A83-01, 500 nM (PeproTech, Cranbury, NJ, USA, 9094360); and NA 10 nM (Sigma-Aldrich, St. Louis, MO, USA, N0636). Medium was changed every 2 to 3 days. Organoids were passaged every 7 to 10 days by detaching Matrigel drops from the wells (with the pipette), pipetting up and down vigorously to ensure disaggregation, and centrifuging (600 g for 6 min) to break up the organoids and remove Matrigel. Pellet was resuspended in advanced DMEM/F12-Matrigel as described above.

### 2.3. EndoECM-Hydrogel-Based Culture

Porcine uterine EndoECM hydrogel [30] was used in this study. Briefly, uterine horns (*n* = 5) recovered from the local slaughterhouse (Mercavalencia, Valencia) were cannulated and decellularized following a previously established protocol [31]. The endometrial fraction of the acellular tissue was isolated by microdissection under a stereomicroscope (Nikon, Leuven, Belgium, SMZ800) and subjected to additional DNA digestion and washing. Subsequently, endometrial tissue was milled in a mortar with liquid N2, lyophilized, pepsin-digested (Sigma-Aldrich, St. Louis, MO, USA, P7000), and neutralized using a modified protocol [32]. The resulting EndoECM pre-gel solution was stored at −80 °C. EndoECM was characterized at different stages of the decellularization process (isolated DC endometrium, lyophilized endometrial powder, and EndoECM) [30].

For culture media supplementation, thawed EndoECM pre-gel solution was added to prewarmed ExM at a final concentration of 0.1 mg/mL. This addition occurred immediately before every change of medium. To avoid polymerization of the hydrogel and ensure that ECM components remained solubilized, vigorous pipetting was applied.

Early passage organoids (p5) from three different biopsies were cultured over 7 days in four different culture conditions (Table S1). ExM was set as a control group, while NA depletion (ExM-NA) was used as a suboptimal growth condition. Both media were supplemented with soluble EndoECM as described above to generate the ExM+EndoECM and ExM-NA+EndoECM conditions.

### 2.4. Histological and Immunohistochemical Characterization of Organoids

Matrigel drops were dissolved by adding cell recovery solution (Corning™, Bedford, MA, USA, 354253) directly into the wells for 60 min at 4 °C. After that, organoids were collected, transferred to an Eppendorf tube, and centrifuged (600× *g* for 6 min). To fix organoids, 4% formaldehyde (Sigma-Aldrich, St. Louis, MO, USA, 1.00496) was added to organoid pellets for 30 min at 4 °C. Subsequently, three cycles of centrifugation and resuspension in 1 mL PBS 1X (Sigma-Aldrich, St. Louis, MO, USA, P3813) were performed to mix the organoids with 200 µL HistoGel (Thermo Fisher Scientific, Kalamazoo, MI, USA, HG-4000-012) preheated at 55 °C. Sample blocks were dehydrated, embedded in paraffine, and cut in 4 μm sections. Hematoxylin and eosin (H&E) and PAS (Sigma-Aldrich, St. Louis, MO, USA, 395B) staining were performed according to the manufacturer’s protocol to visualize glandular structures and specific secretions such as glycogen. For immunohistochemistry and immunofluorescence, tissue sections were deparaffinized, rehydrated, and subjected to heat-induced epitope retrieval (HIER) with 10 mM pH 6 sodium citrate buffer for 20 min at 95 °C in a water bath. Expressions of MUC-1 and Ki67 were evaluated by immunohistochemistry. For MUC-1, samples were permeabilized with PBS–Tween 0.05% and blocked with PBS–BSA 5%–Tween 0.05% for 1 h at room temperature (RT), and primary antibody anti-MUC-1 (Abcam, Cambridge, UK, ab109185, 1:250) was incubated overnight at 4 °C. For Ki67, permeabilization was performed with PBS–Triton 0.4%, and samples were blocked with PBS–BSA 5%–Tween 0.05% for 90 min at 37 °C. Primary antibody anti-Ki67 (Dako, Agilent Technologies, Glostrup, Denmark, M7240, 1:100) incubation was performed at RT for 20 min. After MUC-1 and Ki67 incubations, endogenous peroxidase activity was blocked with 3% H_2_O_2_ for 10 min at RT, and slides were incubated with labeled-polymer HRP for 30 min. Subsequently, slides were incubated with substrate–chromogen for 5 min and counterstained with hematoxylin. Expressions of pan-cytokeratin and vimentin were assessed by immunofluorescence. Samples were permeabilized with PBS–Tween 0.05% and blocked with PBS–BSA 5%–Tween 0.05% for 1 h at RT. Incubation of primary antibody anti-PanCK (Abcam, Cambridge, UK, ab86734, 1:100) was performed overnight at 4 °C, and secondary antibody Alexa Fluor 488 goat anti-mouse IgG1 (Invitrogen, Eugene, OR, USA,, A21121, 1:500) was incubated for 45 min at RT. After that, primary antibody anti-vimentin (Abcam, Cambridge, UK, ab92547, 1:250) was incubated for 30 min RT, followed by secondary antibody Alexa Fluor goat anti-rabbit 555 (Invitrogen, Eugene, OR, USA, A21429, 1:500) incubation for 45 min RT and mounting with DAPI (Thermo Scientific, Rockford, IL, USA, 62248). Tissue sections were imaged using a Nikon Eclipse 80i microscope (Nikon, Leuven, Belgium) and processed with ImageJ software [33].

### 2.5. Chromosomal Stability

Organoids exposed to ExM medium from three different biopsies at early (p3) and late (p8) passages were analyzed. Chromosomal stability in p5 organoids exposed to ExM+EndoECM medium was also analyzed (*n* = 3). DNA was extracted from organoids using a Cells and Tissue DNA Isolation Micro Kit (Norgen, Thorold, ON, Canada, 57300). DNA quality and concentration were measured using the NanoDrop ND-1000 Spectrophotometer, and its integrity was evaluated by electrophoresis on a 0.8% agarose gel to verify the presence of a band around 10–20 kb. Chromosomal stability was analyzed using the genomewide high-resolution Affymetrix Cytoscan 750K array (Affymetrix Inc., Santa Clara, CA, USA). The array procedure was performed according to the manufacturer’s recommendation. Microarray data were processed and analyzed using Affymetrix Chromosome Analysis Suite (ChAS 4.2) in combination with a referenced model provided by the manufacturer (hb38).

### 2.6. Comparative Proliferation Assays

Passage 5 organoids were cultured for 7 days under the four experimental conditions described above (ExM, ExM+EndoECM, ExM-NA, ExM-NA+EndoECM). Twelve drops were cultured and analyzed per biopsy (*n* = 3) and condition. To avoid variations in the initial concentration of organoid precursors, samples were pooled and seeded alternately between all conditions. On days 2, 5, and 7 of culture, photos of all wells (12 per condition) were taken with an inverted Zeiss Axio Vert.A1 microscope (Zeiss, Oberkochen, Germany) at ×5 magnification. The number of spheroids was counted manually. To avoid bias, the counting was carried out in triplicate by three independent observers.

Similarly, the proliferation data exhibited by Ki67 immunostaining were obtained by counting positive and total cell nuclei in five photos per biopsy (*n* = 3) and condition (ExM, ExM-NA, ExM+EndoECM, and ExM-NA+EndoECM) and compared. Two independent analyses were performed by blind counting comparison.

### 2.7. Stemness Assessment

Expression of typical epithelial progenitor cell markers in organoids was assessed by immunofluorescence. Sections were permeabilized with PBS–Tween 0.05% and blocked with PBS–BSA 5%–Tween 0.05% for 1 h at RT. Incubation of primary conjugated antibodies anti-SSEA-1 (Bioss Inc., Woburn, MA, USA, bs-1702R-A594, 1:50) and anti-N-cadherin (Bioss Inc., Woburn, MA, USA, bs-1172R-A488, 1:50) was performed for 1 h at RT, followed by incubation with DAPI. Tissue sections were imaged using a Nikon Eclipse 80i microscope and processed with ImageJ.

### 2.8. Statistical Analyses

Quantitative data are expressed as mean value ± standard deviation (SD). Having verified the normal distribution of the data, ANOVA and Tukey’s range tests were used to make comparisons between the different culture conditions. The data were analyzed using SPSS (SPSS, Inc., Chicago, IL, USA). A *p*-value ≤ 0.05 in a two-tailed test was considered statistically significant.

## 3. Results

### 3.1. Human Endometrial Organoids Cultured with EndoECM Supplementation Recapitulate the In Vivo Phenotype of Endometrial Glands

**Figure 2:**
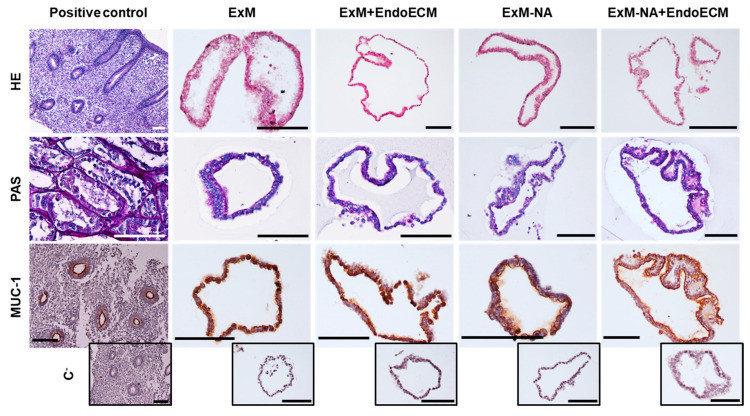
Characterization of glandular origin in human endometrial organoids by histological and immunohistochemical staining. H&E, PAS staining, and MUC-1 expression in positive controls (endometrium and kidney) and in human endometrial organoids cultured in the four different experimental conditions: ExM, ExM+EndoECM, ExM-NA, ExM-NA+EndoECM. Negative controls for MUC-1 are shown at the bottom of the figure in small size. Scale bars are 100 µm.

To assess the organoids’ structure and confirm their glandular origin, H&E staining and the expression of glandular endometrium characteristic markers (MUC-1, glycogen) was evaluated in organoids across all culture conditions (Figure 2). H&E organoid staining corroborated the original histological characteristics of the epithelial tissue. PAS staining revealed glycogen secretion by the epithelial cells of the organoids, an important component of glandular secretions observed in endometrial glands in vivo. MUC-1 was strongly expressed and secreted into the luminal compartment of the organoids, comparable to luminal glandular epithelial cells of human endometrium. While the main concern of the study was the biosuitability of porcine EndoECM for culturing human organoids, this evidence showed that organoids could develop efficiently in the presence of the matrix.

**Figure 3:**
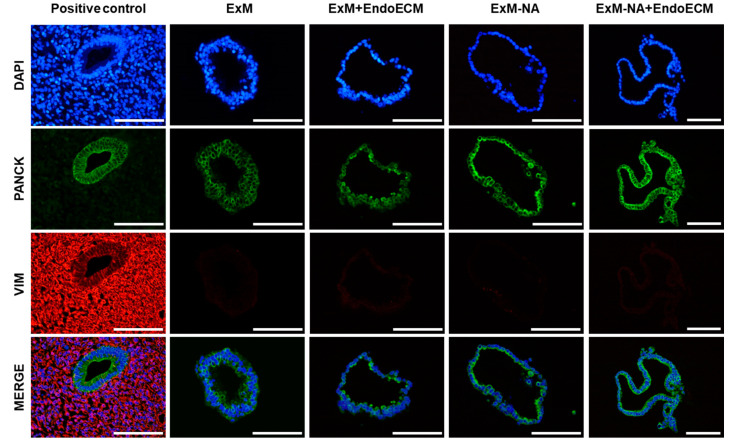
Characterization of epithelial phenotype in human endometrial organoid by immunofluorescence staining. Epithelial (pan-cytokeratin) and stromal (vimentin) marker expressions in human endometrium and organoids cultured in the four different experimental conditions: ExM, ExM+EndoECM, ExM-NA, and ExM-NA+EndoECM. Cell’s nuclei stained with DAPI. Scale bars are 100 µm.

Expression of pan-cytokeratin and vimentin was evaluated to demonstrate the enrichment of epithelial cells over stroma during organoid development in the four different culture conditions (Figure 3). Pan-cytokeratin localized in the cytoplasmic compartment of organoid cells, but vimentin was absent, corroborating the epithelial origin of the organoids in all the experimental conditions.

Histological and immunohistochemical assays did not reveal any significant differences between the four experimental conditions (ExM, ExM+EndoECM, ExM-NA, ExM-NA+EndoECM) (Figure 2 and Figure 3). Addition of EndoECM pre-gel solution into the expansion medium did not modify the glandular epithelial marker expression, leading to the maintenance of an in vivo-like phenotype in the 3D structures throughout the culture period.

### 3.2. Human Endometrial Organoids Are Genetically Stable during Long-Term Culture Systems

**Figure 4:**
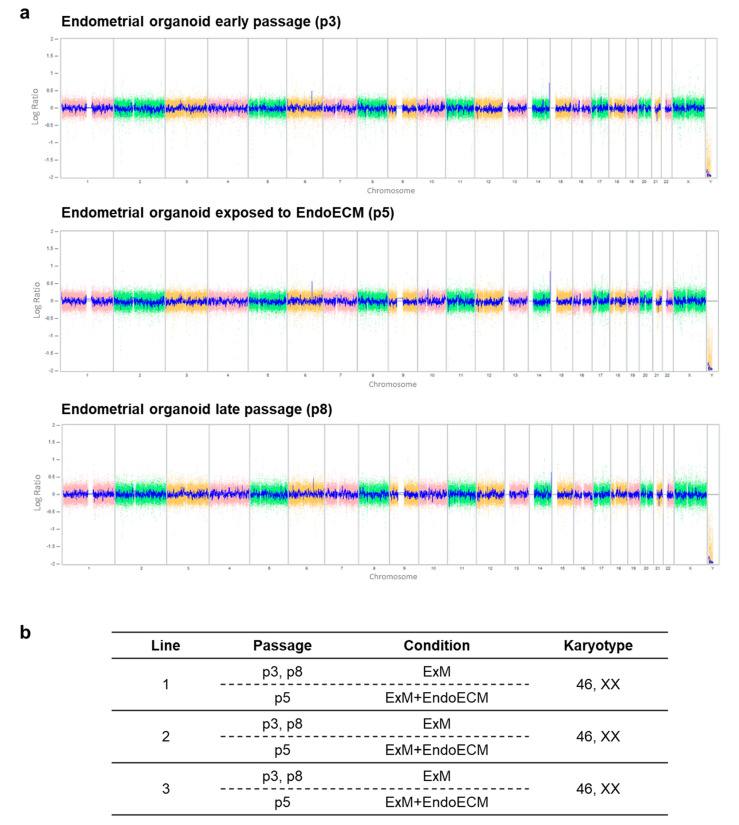
Chromosomal analysis of human endometrial organoids by cytogenetic microarray. (**a**) Genomic DNAs from early (p3), mid (p5), and late (p8) passage organoids exposed to ExM and EndoECM culture conditions are compared with a human reference genome. Intensity of each probe (log ratio) is expressed on the Y-axis and its location on chromosomes (1–22, X and Y) on the X-axis. A signal ratio of 0 indicates copy number equivalence. Reduced signal on the Y chromosome is not significant due to the absence of this chromosome in the samples. (**b**) Summary of genetic analyses.

The chromosomal stability of endometrial organoids exposed to ExM in p3 and p8 and organoids supplemented with EndoECM in p5 was evaluated using a cytogenetic microarray. For each established organoid line exposed to ExM medium, genomic DNAs at early (p3) and late (p8) passages were compared with a reference genome. No DNA copy number alterations were detected after more than 2 months of continuous passage (Figure 4). Further, exposure of p5 organoids to ExM+EndoECM had no effect on chromosomal stability. All established organoid lines showed a normal 46, XX karyotype.

### 3.3. Tissue-Specific Extracellular Matrices Support Organoid Proliferation

#### 3.3.1. Exposure of Human Endometrial Organoids to EndoECM Had a Positive Effect on Their Proliferation Rate

**Figure 5:**
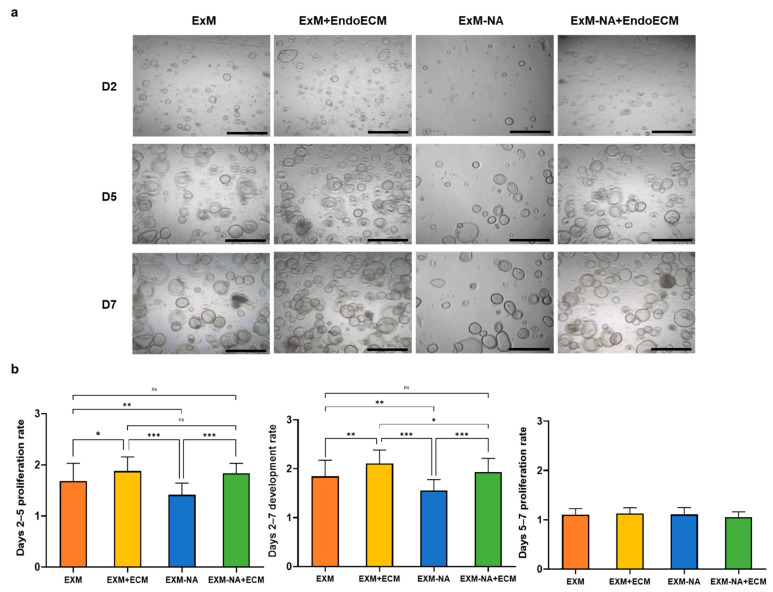
Comparative proliferation of human endometrial organoids exposed to different conditions. (**a**) Representative images of endometrial organoids in all culture conditions: ExM, ExM+EndoECM, ExM-NA, ExM-NA+EndoECM. (**b**) Days 2–7, 2–5, and 5–7 proliferation rates. * *p* < 0.05, ** *p* < 0.001, *** *p* < 0.0001. Scale bars are 1 mm.

To evaluate the effect of EndoECM components on organoid development, standard (ExM) and NA-deficient culture media were supplemented with EndoECM pre-gel solution. Organoids were counted on days 2, 5, and 7 of culture. Significant organoid formation and development was observed in all conditions (Figure 5a). The ExM+EndoECM group showed higher day 2–7 proliferation rates compared with the organoid group exposed to only ExM (2.11 ± 0.27 vs. 1.84 ± 0.33, respectively, *p* < 0.001). Likewise, significant differences in proliferation were observed in days 2–7 between the NA-deficient groups, with a higher proliferation rate in the EndoECM-supplemented group (1.94 ± 0.28 and 1.55 ± 0.22 for ExM-NA+EndoECM and ExM-NA groups, respectively, *p* < 0.0001). Therefore, EndoECM not only was able to improve standard culture conditions in terms of proliferation, but also made up for deficiencies of the medium (absence of NA). Day 2–5 proliferation rates showed similar results to days 2–7, with higher proliferation rates in the ExM+ECM group compared with organoids exposed to ExM. Day 5–7 proliferation rates were practically the same for all conditions (Figure 5b). This finding indicates that on day 5 of culture, few new organoids are formed, but existing organoids can maintain growth and differentiation characteristics.

#### 3.3.2. Human Endometrial Organoids Cultured under EndoECM Supplementation Show Higher Proliferation Capacity Based on Ki67 Expression

**Figure 6:**
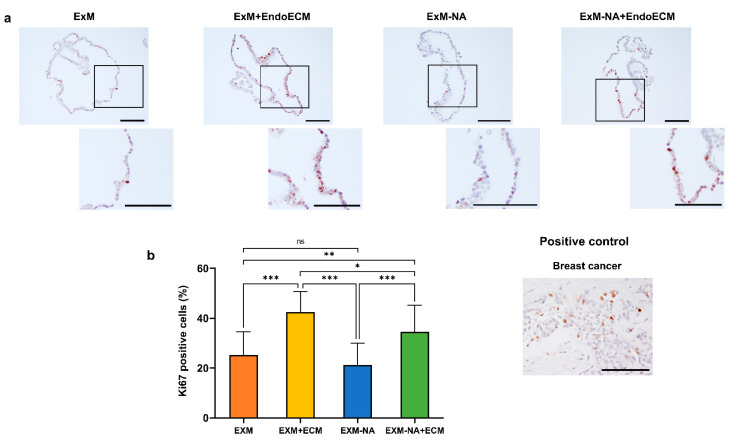
Human endometrial organoids proliferation assay. (**a**) Immunohistochemistry for Ki67 expression in breast cancer and organoids cultured in the four different experimental conditions ExM, ExM+EndoECM, ExM-NA, ExM-NA+EndoECM. (**b**) Percentage of Ki67 positive cells in organoids. * *p* < 0.05, ** *p* < 0.01, *** *p* < 0.0001. Scale bars are 100 µm.

To determine the effect of EndoECM addition and NA withdrawal in the culture media (ExM) on organoid proliferation capacity, Ki67 expression was evaluated by immunohistochemistry in the four experimental conditions: ExM, ExM+EndoECM, ExM-NA, and ExM-NA+EndoECM (Figure 6a). Ki67 expression was observed in every condition.

Ki67 proliferation was calculated from the number of stained nuclei versus the total number of nuclei. Culture conditions could be divided into three significantly differentiated groups (*p* < 0.05) (Figure 6b): Organoids cultured in the EXM supplemented with EndoECM showed the highest proliferative capacity (42.47 ± 8.2%) compared with other culture conditions, followed by those developed without NA but with EndoECM addition (34.61 ± 10.6%). ExM and ExM-NA conditions showed less proliferation activity (25.2 ± 9.3% and 21.25 ± 8.8%, respectively). These results confirm the positive effect of EndoECM on the proliferation rate observed by counting the number of spheroids.

**Figure 7:**
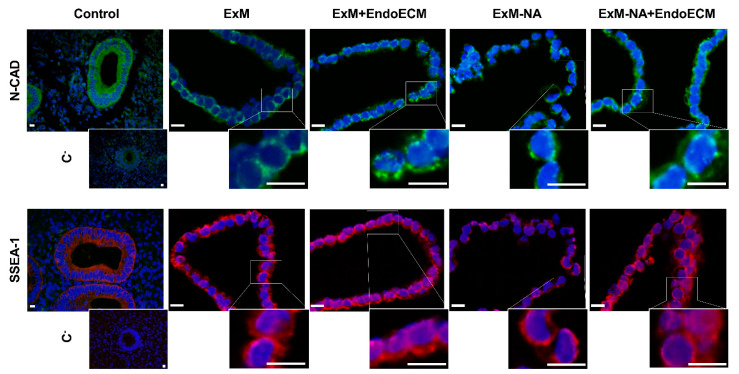
Epithelial progenitor cell markers in human endometrial organoids. N-cadherin (green) and SSEA-1 (red) expressions in positive controls (human endometrium) and human endometrial organoids cultured in the four different experimental conditions: ExM, ExM+EndoECM, ExM-NA, ExM-NA+EndoECM. A higher magnification of the boxed areas is shown in all panels. Cell’s nuclei stained with DAPI. Scale bars are 10 µm.

The stemness characteristics of the organoids, closely related to their clonogenic features, was tested through the detection of N-cadherin and SSEA-1, two typical epithelial progenitor cell markers. Both markers were detected ubiquitously in all organoid cells, with no differences between culture conditions (Figure 7).

## 4. Discussion

We describe a bioengineering approach to optimize genetically stable human endometrial organoid culture by supplementing the culture medium with an ECM hydrogel solution derived from decellularized porcine endometrium [30]. Organoids not only grew and recapitulated the molecular and functional characteristics of the tissue of origin, but also showed a higher proliferation capacity than those cultured by previously described techniques [14,15,16].

The lack of well-established experimental models of uterine diseases such as endometriosis, adenomyosis, Asherman’s syndrome, and endometrial atrophy makes for incomplete evaluation of their pathogenesis and limits the development of effective treatments to improve fertility in patients with these conditions [34]. Organoids have been reported as disease culture systems to study pathologies since they mimic the in vivo organ from which they are derived [35]. However, the 3D system does not reside in the tissue-specific ECM, whose physicochemical features play a crucial role in homeostasis. For example, it has been postulated how possible defects in the endometrium and specifically in the ECM remodeling can give rise to the development of endometriotic lesions. Among others, the extracellular matrix metalloproteinases alter the peritoneal microenvironment, creating the conditions for differentiation, adhesion, proliferation, and survival of ectopic endometrial cells [36,37].

To fully model the mechanical properties, tissue-specific signaling cues, and bioactive components of the ECM in vitro, tissue decellularization techniques have grown in popularity [31,38,39,40,41,42,43]. Since the first publication in the 1990s, ECM hydrogels have been generated from virtually all human tissues and organs [28,29,30,44,45,46,47,48,49], and their putative use in in vitro assays has been reported widely [50]. Recently, our group developed the first endometrial ECM hydrogel of porcine origin (EndoECM), and having tested its cytocompatibility, we hypothesized that a natural ECM derived from decellularized endometrium could render a physiologically relevant native environment, optimizing in vitro culture systems of human endometrial organoids.

Our findings suggest that endometrial organoids exposed to EndoECM reproduce the glandular epithelial characteristics of the native tissue just like organoids derived with normal expansion medium. Immunohistochemistry highlighted the expressions of several cytokeratins (1, 4, 5, 6, 8, and 18 intermediate filament proteins), which play important roles in maintaining the structural integrity of epithelial cells and are crucial in differentiation, tissue specialization, and function [51]. In addition, organoids derived in the presence of EndoECM produced glycogen, the major component of endometrial glandular secretions, and MUC-1 (mucus production) directly into the luminal compartment. Based on these findings, we suggest that our organoids mimic the functional behavior of glandular tissue previously described in endometrial organoids [14], including glandular-type organization and expressions of specific markers [15]. Further, our results suggest that endometrial organoids can be expanded in the long term without accumulating chromosomal alterations when exposed to EndoECM and can maintain the phenotype of in vivo endometrial glands, avoiding the limitations of 2D cell cultures.

Moreover, endometrial organoids were maintained under long-term culture in a medium that contains activators of WNT signaling (RSPO1) and growth factors (EGF, HGF, and FGF10) to promote proliferation. Inhibitors of TGFβ and BMP signaling pathways (A83-01 and Noggin, respectively) were added to prevent differentiation, as well as NA for the establishment of organoids [14,15,52]. These culture conditions promote enrichment in clonogenic and self-renewing epithelial cells with high proliferative potential, key properties of human endometrial stem/progenitor cells [53,54,55]. This could explain the observed expression of typical epithelial progenitor markers N-cadherin and SSEA-1 in most organoid cells under different culture conditions.

Cell survival, proliferation, and differentiation are controlled by bidirectional signaling between cells and the surrounding milieu. These processes are mediated by ECM proteins such as laminin, collagen, and fibronectin binding to transmembrane integrin receptors, which are activated by cytoskeletal components [56]. In our study, when endometrial organoids were exposed to EndoECM pre-gel solution, which is highly enriched in extracellular proteins and factors (collagens, fibronectin, dermatopontin, azurocidin, fibrinogen, or extracellular kinases) [30], we observed the generation of a higher number of spheroids in comparison with standard culture conditions. In addition, EndoECM supplementation counteracted the negative effects of NA withdrawal on organoid formation. NA is the main precursor of nicotinamide adenine dinucleotide (NAD+), a coenzyme essential for DNA repair, glycolysis, and oxidative phosphorylation [57]. Although its underlying mechanisms of action are unclear, NA has previously been shown to improve organoid culture efficiency, being essential for the establishment and/or long-term culture maintenance of epithelial organoids because it promotes cell survival and differentiation [14,58]. Our results demonstrate that organoids exposed to culture media without NA but supplemented with tissue-specific EndoECM had a higher proliferation rate than organoids exposed to ExM without NA and similar proliferation to organoids with NA and supplemented with EndoECM, suggesting that EndoECM supplementation could facilitate complex cellular behavior even without NA present. Proliferation assays based on Ki67 expression confirmed these results; independent of the presence of NA, organoids exposed to EndoECM had a higher proliferation rate. Based on these findings, organoid culture medium supplemented with hydrogel derived from a decellularized endometrium (EndoECM) could be an optimal platform for in vitro organoid development, improving on common standard approaches, such as the use of Matrigel alone, which is unable to recapitulate the in vivo dynamic changes. It should be noted that the reduced sample size of the study could be a limitation, this work being a proof of concept. Related to the effect of the hormonal profile from female donors might have on our results, the same starting material was used for all experimental conditions, so that the relative comparisons are to some extent standardized.

Although Matrigel is commonly used as scaffold in 3D cultures [59,60,61], it has some limitations due to its animal origin and chemically undefined properties. The use of Matrigel could be disadvantageous for high throughput screening, and its direct transplantation could be limited by current medical legislation [10]. Furthermore, depending on the characteristics, architecture, and associated pathology, in vivo tissues exhibit physical properties that are found to be anisotropic, nonlinear, and heterogeneous [20]. Advantageously, hydrogels derived from decellularized endometrium recapitulate tissue-specific composition, signaling, and dynamic changes, improving organoid proliferation rates as we have demonstrated here. Although their animal origin could be a limitation because it could imply batch-to-batch variation, this issue has not stopped the Food and Drug Administration (FDA) from approving many decellularized tissue-specific ECM for human uses [62]. In our case, EndoECM hydrogel from the same stock and experiment was used throughout the study. However, to fully replace Matrigel with tissue-specific scaffolds that reliably reproduce EndoECM characteristics, we would need to enhance the stability and mechanical properties of our EndoECM hydrogels. To this end, the use of chemical crosslinking [63] or a semi-synthetic mixture with more stable compounds [64,65] will be investigated.

Lack of stroma, blood vessels, innervation, and immune cells is also a limitation to organoid research despite EndoECM supplementation. Organoid and stromal cell coculture is an emerging technique that aims to reproduce epithelial–stromal interactions [66]. Coculture of human endometrial organoids with stromal cells could facilitate the study of epithelium–stroma interactions [17]. Further, endometrial organoids made of both primary epithelial and stromal cells have been developed, although their expandability is limited because they cannot be passaged efficiently [67,68]. This approach, combining current organoid culture methods with the use of EndoECM, could result in a useful platform for investigating the cell types and molecular pathways involved in remodeling the endometrium and how deregulation of these mechanisms may lead to disease development.

In summary, supplementation of organoid culture medium with hydrogel derived from decellularized porcine endometrium mimics the biochemical aspects of the endometrial tissue, giving rise to a specific cellular microenvironment permitting organoid development with good cell viability, direct cell adhesion, differentiation, and increased proliferation. This study is a step forward in the generation of stable models that faithfully reproduce the physiology and pathology of the human endometrium. Matrix–cell interactions and remodeling or adhesion events are increasingly close to being studied in vitro.

## 5. Conclusions

We demonstrated the importance of mimicking a favorable native microenvironment for the development of human endometrial organoids. Supplementation of the culture medium with a pre-gel solution of EndoECM is biocompatible and enables the growth, function, and phenotype of endometrial organoids while also enhancing their proliferative potential. While further research is needed to develop a platform based exclusively on ECM, hydrogels based on this material are an optimal platform for in vitro organoid development, improving on standard approaches.

## Data Availability

Not applicable.

## Supplementary Materials

The following are available online at https://www.mdpi.com/article/10.3390/jpm11060504/s1: Table S1: Culture media composition.
