# Supplementary material for: Improved Models of Human Endometrial Organoids Based on Hydrogels from Decellularized Endometrium
Source: J Pers Med. 2021 Jun 3;11(6):504. doi: 10.3390/jpm11060504 (PMC8229407; doi:10.3390/jpm11060504)
Supplement: Supplementary file 1 [file jpm-11-00504-s001.zip › jpm-1185814-supplementary.pdf]

Table S1. Culture media composition

Component	Final Concentration	ExM	ExM+EndoECM	ExM-NA	ExM-NA+EndoECM
Advanced DMEM/F12	1X	x	x	x	x
N2 supplement	1X	x	x	x	x
B27 supplement minus vitamin A	1X	x	x	x	x
Primocin	100 µg/mL	x	x	x	x
N-Acetyl-L-cysteine*	1.25 mM	x	x	x	x
L-glutamine	2 mM	x	x	x	x
Recombinant human EGF	50 ng/mL	x	x	x	x
Recombinant human Noggin	100 ng/mL	x	x	x	x
Recombinant human Rspondin-1	500 ng/mL	x	x	x	x
Recombinant human FGF-10	100 ng/mL	x	x	x	x
Recombinant human HGF	50 ng/mL	x	x	x	x
ALK-4, -5, -7 inhibitor, A83-01	500 nM	x	x	x	x
Nicotinamide	10 nM	x	x		
EndoECM	100 µg/mL		x		x

ExM: Expansion medium; EndoECM: endometrial extracellular matrix; NA; Nicotinamide; DMEM: Dulbecco's Modified Eagle's medium; EGF: Epidermal growth factor; FGF: Fibroblast growth factor; HGF: Hepatocyte growth factor; ALK: Anaplastic lymphoma kinase.
